# Clinical features of familial HAM/TSP

**DOI:** 10.1186/1742-4690-11-S1-O7

**Published:** 2014-01-07

**Authors:** Satoshi Nozuma, Eiji Matsuura, Toshio Matsuzaki, Osamu Watanabe, Ryuji Kubota, Shuji Izumo, Hiroshi Takashima

**Affiliations:** 1 Department of Neurology and Geriatrics, Kagoshima University Graduate School of Medical and Dental Sciences, Kagoshima City, Kagoshima, Japan; 2 Department of Molecular Pathology, Center for Chronic Viral Diseases, Kagoshima University, Japan

## 

Some genetic factors are associated with the development of HTLV-1-associated myelopathy/tropical spastic paraparesis (HAM/TSP). So far, there is a very few report about familial HAM/TSP. This study aimed to clarify the clinical features of familial HAM/TSP. We reviewed all patients with HAM/TSP, 784 in number, hospitalized to Kagoshima University Hospital from 1987 to 2012. Familial HAM/TSP cases (patients with one or more HAM/TSP patients in their family) were compared with 124 sporadic HAM/TSP cases (patients with no other HAM/TSP patient in their family) admitted in series for association of clinical features in an unmatched case-control design. As a result, 40 patients (5.1%) in total 784 were familial cases. In familial cases compared to sporadic cases, age of onset was earlier (41.3 year old vs. 51.6 year old, P<0.001), the number of acute progression cases was smaller (10.0 percent vs. 28.2 percent, p=0.019), motor disability grade was lower (4.0 vs. 4.9, p=0.043) in spite of longer duration of illness (14.3 years vs. 10.2 years, P=0.026), and duration between onset and time to use a wheelchair in daily life was longer (18.3 years vs. 10.0 years, P=0.025) significantly. Protein in cerebrospinal fluid (CSF) was significantly lower in familial cases (29.9 mg vs. 42.5 mg, p<0.001). HTLV-1 provirus load, anti-HTLV-1 antibody in serum and CSF, cells in CSF was not significantly different. Thus, we demonstrate familial HAM/TSP showing younger onset and slower progress than in sporadic cases. Our results suggest that some genetic factors might influence the incidence of familial HAM/TSP.

